# Multisensory Flavour Perception: Blending, Mixing, Fusion, and Pairing within and between the Senses

**DOI:** 10.3390/foods9040407

**Published:** 2020-04-01

**Authors:** Charles Spence

**Affiliations:** Crossmodal Research Laboratory, Oxford University, Oxford OX2 6GG, UK; Charles.spence@psy.ox.ac.uk

**Keywords:** complexity, blending, fusion, confusion, mixing, flavour perception, flavour pairing

## Abstract

This review summarizes the various outcomes that may occur when two or more elements are paired in the context of flavour perception. In the first part, I review the literature concerning what happens when flavours, ingredients, and/or culinary techniques are deliberately combined in a dish, drink, or food product. Sometimes the result is fusion but, if one is not careful, the result can equally well be confusion instead. In fact, blending, mixing, fusion, and flavour pairing all provide relevant examples of how the elements in a carefully-crafted multi-element tasting experience may be combined. While the aim is sometimes to obscure the relative contributions of the various elements to the mix (as in the case of blending), at other times, consumers/tasters are explicitly encouraged to contemplate/perceive the nature of the relationship between the contributing elements instead (e.g., as in the case of flavour pairing). There has been a noticeable surge in both popular and commercial interest in fusion foods and flavour pairing in recent years, and various of the ‘rules’ that have been put forward to help explain the successful combination of the elements in such food and/or beverage experiences are discussed. In the second part of the review, I examine the pairing of flavour stimuli with music/soundscapes, in the emerging field of ‘sonic seasoning’. I suggest that the various perceptual pairing principles/outcomes identified when flavours are paired deliberately can also be meaningfully extended to provide a coherent framework when it comes to categorizing the ways in which what we hear can influence our flavour experiences, both in terms of the sensory-discriminative and hedonic response.

## 1. Introduction

### 1.1. Searching for Novelty and Interest in Cuisine

Combining different elements in our food and drink experiences is commonly considered to deliver a desirable result—otherwise, why bother? However, above and beyond the mundane combining of different ingredients that one normally finds in everyday recipes [1], it is also a desirable practice insofar as it may help to deliver a novel result, as is the case in so many contemporary fusion foods, [2]. It would appear that many diners and consumers today, as previously [3,4,5,6], crave novelty in their cuisine. One of my favourite early quotes highlighting that the craving for novelty should not be considered solely a contemporary passion comes from the famous chef Auguste Escoffier (head cook of the Paris Ritz and London Savoy). A little over a century ago, he wrote that: “It is an exceedingly common mania among people of inordinate wealth to exact incessantly new or so-called new dishes… Novelty! It is the prevailing cry; it is imperiously demanded by everyone.… What feats of ingenuity have we not been forced to perform, at times, in order to meet our customer’s wishes? Personally, I have ceased counting the nights spent in the attempt to discover new combinations.” [7] (p. vii). Indeed, as Visser [8] (p. 124) presciently noted some 30 years ago, the contemporary taste for novelty offers “a wonderful marketing milieu”. Novelty in the world of food and drink has traditionally been delivered by sourcing new ingredients or culinary techniques, or else by introducing the cuisine of an exotic, and preferentially unfamiliar, region/culture. However, by mixing flavours, ingredients, and/or culinary techniques in novel ways one can potentially also deliver successful new fusion foods, as evidenced by the dramatic rise in popularity of novel pastry items such as the ‘cronut’ [9,10,11], and its many imitators, including the ‘mufgel’ [12] and the ‘croiffle’ [13]. Here, though, it is worth noting that the popularity of a number of these new fusion foods can presumably also be put down to their Instagrammability [14,15].

Combining distinct flavours/elements in a multisensory tasting experience must result in a more complex outcome, chemically-speaking [16]. It has often been suggested that increased complexity is a desirable attribute as far as many flavour/product experiences are concerned (e.g., in the case of wine; [17,18,19]). As wine-maker, Josh Jensen puts it: “When you move up the quality scale, it isn’t that the wines are more powerful or riper tasting, or more oaky. It’s that they have more levels, more nuances. It’s really all about complexity of flavour.” [20] (p. 284). That said, the extent to which that complexity is perceivable by the consumer is, I would argue, open to debate [21,22,23,24,25].

At one level, the majority of food and drink products that we tend to consume on a daily basis already involve the combination of different elements, be they ingredients, flavours, or culinary techniques. However, the end result (or aim) of that combination may either be to blend similar flavours/ingredients/techniques, or else to mix dissimilar ones. It is important to note here that the combination of different elements in a dish, drink, or food product can give rise to a range of different outcomes, including everything from fusion, in the more successful cases [2,11], through to confusion in the rather less successful ones [26,27].

### 1.2. Outline

In this narrative review, I want to look at the various ways in which different elements are combined in the design of food and drink experiences from both the chef/bar perspective and from the commercial products/experiences (e.g., as in the case of flavour pairing) that have increasingly been introduced into the marketplace in recent years. This review is separated into two main parts. First, I briefly review the literature on the perceptual consequences of blending and mixing [28], as well as highlighting the continued/increasing popularity of fusion foods [2,11] and flavour pairing [29,30,31]. One of the questions that will be discussed concerns whether there are any rules/guidelines that may help those wanting to deliver novel flavour experiences/food products by combining more or less familiar elements in future food experiences [30,31]. In the latter part of the review, I will then take the findings regarding the pairing of flavours and consider whether the same perceptual outcomes may provide a meaningful framework for understanding what happens when flavours/foods are deliberately paired with particular music/soundscapes. Importantly, I suggest that many of the various perceptual pairing principles/outcomes identified when thinking about what happens when flavours are deliberately paired can be meaningfully extended to provide a coherent framework when it comes to categorizing the ways in which what we hear can influence our flavour experiences, both in terms of the sensory-discriminative and hedonic response of consumers. At this stage of development of the field of pairing research involving flavour stimuli, a narrative review would seem more appropriate rather than either a systematic review or meta-analysis. It should, however, be noted that thorough reviews of many of the individual sub-topics can be found in the relevant review papers cited within the text. 

## 2. Blending and Mixing

The results of a number of studies that have been conducted over the last half-century or so have demonstrated that neither experts nor non-experts appear able to unpick blends, be they blends of single varietal grapes/wines or whiskies, in order to identify their contributing components [32,33,34,35]. At the outset here, one might be tempted to wonder what exactly differentiates blending from mixing. As a rule of thumb, blending would appear to refer to the deliberate combination of various expressions of the same product, be it grapes, whiskies, or tea leaves [36,37]. When sound artists start talking of the new field of ‘oenesthesia’ as the blending of wine with music, they are presumably trying to stress the correspondence, or metaphorical similarity, between the wine and the carefully composed matching music [38].

### 2.1. Wine Blends

Harrar et al. [33] conducted a study involving 15 tasters, comprising four experts, six intermediates, and five novice Champagne tasters, in which seven different sparkling wines were presented blind. The participants were only informed that the sparkling wines (six Champagnes and one English sparkling wine) could potentially span the full range from 0% to 100% Chardonnay white grapes but were otherwise given no information about what they were tasting. Furthermore, all visual cues that might have helped the participants were also deliberately obscured with the sparkling wines presented in identical opaque black tasting glasses. The tasters were instructed to try and estimate the proportion of white (i.e., Chardonnay) grapes in each of the wines while, at the same time, also rating their hedonic response to each wine. The sparkling wines varied systematically from a 100% Blanc de Blancs (made with 100% white Chardonnay grapes) through to a 100% Blanc de Noirs (made with 100% red Pinot Noir and/or Pinot Meunier grapes; the exact percentages of white Chardonnay grapes being 0%, 22%, 30%, 45% × 2, 58%, or 100%).

Crucially, however, none of the participants were able to correctly judge the percentage of white grapes in the wines. What is more, the tasters’ hedonic ratings of the wines did not appear to correlate with the price of the sparkling wines either, this despite the fact that they varied from £18–400 a bottle. Here, though, it is important to note that these results do not, in any meaningful sense, imply (as much of the newspaper coverage surrounding this work seemed to want to suggest, at least in the popular press) that the tasters were unable to discriminate between the wines, as a clear hedonic preference for one of the mid-priced Champagnes was, on average, expressed by the participants. Results such as these, therefore, suggest that regardless of the level of tasting expertise (i.e., Champagne experts or not), people are simply not able to correctly distinguish the relative proportion of different grape varieties in sparkling wine. However, one of the other reasons for wanting to blend wines is to enhance the perceived complexity of the resulting mixture. Indeed, early research provided grounds for wanting to blend different single varietals, at least in the case of still wines [39]. (Here, of course, it is worth remembering that even single varietal wines, actually constitute separately vinified barrels that have been blended to create a more satisfying grand vin [20], and may also include a few percent of barrels from the previous vintage too.)

Singleton and Ough [39] selected 34 pairs of similar commercially available, single varietal California wines (dry white and dry red table wines) from the 1960 vintage that had been rated similarly in terms of their quality but which presented somewhat different flavours. A 50-50 mix of each pair of wines was presented together with each pair of single varietals. The participants were thus presented with each trio of wines in a random order while being blind as to what exactly they might be tasting. The 10 experienced sensory panelists who took part in this classic study rated each of the wines on a 20-point quality scale. Intriguingly, the quality scores given to the blends were significantly higher than the mean score of the two single varietal wines. What is more, in seven out of the 34 cases, the blend was actually rated as higher in quality than the best of the two individual components. Singleton and Ough’s suggestion was that the enhanced quality ratings for the blend might have resulted from the increased chemical complexity of the blends. However, that said, it should be stressed that the participants in Singleton and Ough’s study were not asked to rate the perceived complexity of any of the wines that they tasted. Here it is perhaps also worth noting that the notion of perceived complexity in the world of fine wine, is undoubtedly a complex one [18,22,23,24,25]. While some have argued for there being a predictable relationship between the size of monomolecular chemicals and complexity/pleasantness ratings [40,41], the situation is likely to be very different in the case of a quality wine that may contain anywhere between 600–1000 different volatile compounds [42,43].

In a follow-up study inspired by this early research, Wang and Spence [35] assessed whether 87 tasters (41 novice, 30 intermediate, and 16 expert tasters by self-report) were able to detect the chemical complexity of wine by trying to identify the blends from a selection of six wines tasted blind. The wines consisted of three single varietal wines (Cabernet Sauvignon, Merlot, and Cabernet Franc from the Dr Frank Winery, Finger Lakes, NY, USA) and the three possible 50-50 mixtures of each pair of these single varietals. The six wines were presented in a random order. The participants were first requested to rate the perceived complexity, liking, intensity, familiarity, quality, and willingness to pay, as well as listing five flavour descriptors that best matched each wine. Thereafter, they were invited to try and decide which, if any, of the wines that they had just tasted, and still had before them, were blends. The results revealed that none of the three groups of tasters were able to distinguish the presumably more chemically complex [20,25] blends from the single varietals at a level that was significantly better than chance. In fact, for the Cabernet Franc—Merlot pair, the average blend score was actually lower than the lower-scoring wine of the pair. For those who were wondering, the difference in results between the latter two studies (i.e., between [39] and [35]) may say as much about the improvement in wine-making practices over the last half-century or so, as anything else.

### 2.2. Blended vs. Single Malt Whiskies

The story as far as blending grapes for wine has also been replicated in the case of whisky [32,34]. For instance, Chadwick and Dudley had eight Scottish medics taste six whiskies blind (actually blindfolded). The latter were informed that there were three blends (Bells, Haig, and White Horse) and three single malts (Glenfiddich, Springbank, and Glenmorangie) to taste (though without the brands being divulged to the participants), and were tasked with figuring out which of the drinks belonged in each category. They were, in other words, given a sorting, or discrimination task. The whiskies were presented six times giving rise to a total of 36 responses per participant. The whiskies were evaluated in sherry copita glasses containing roughly a single measure to which a thimbleful of spring water had been added. Ref. [44] gives a chemical assessment of the effects of adding water, namely increasing the expression of the smoky guiacol note.

As the authors put it: “Thus, the taste of guiacol and similar compounds will be more pronounced when whisky is further diluted in the glass. This taste-enhancement is counteracted by the dilution of the guiacol concentration. Overall, there is a fine balance between diluting the whisky to taste and diluting the whisky to waste. This balance will depend on the concentration and types of taste compounds that are characteristic for each whisky.” [44] (pp. 7–8). That having been said, it is worth noting that the smoky notes in whisky do not just come from guiacol, as some can also emerge from the roasting of the barrels and the smoking of the peat used to roast the barley.

The results of this admittedly rather informal tasting, which was published as part of the Christmas special issue of the British Medical Journal. revealed little evidence of any ability to discriminate between the blended whiskies and the single malts. In fact, the authors of this study were led to the conclusion that: “the inexpert drinker should choose his whisky to suit his taste and pocket and not his self image” [32] (p. 1913). Such an inability should perhaps not come as such a surprise when it is realised that not everyone can apparently distinguish whisky from cognac under blind tasting conditions either [45]. The latter study had four participants, members of a wine club, take part. The participants tasted two malt whiskies (Glenfiddich and Springbank; i.e., West of Scotland vs. Highland) and two brandies (Courvoisier and Rémy Martin) blindfolded from cut glass tumblers without the addition of any water. The participants had to try and identify first whether the drinks were whisky or cognac, and then try to discriminate the brand, which had been identified prior to the study. The participants tasted the four drinks (approximately) three times before dinner and again after dinner on two separate occasions. The results revealed that one participant performed at chance when answering either of the questions. Two more were approximately 72% and 65% correct on discriminating whisky from brandy (i.e., significantly better than chance) but were essentially at chance as far as identifying the brand was concerned. A number of surprising failures to distinguish been seemingly different-tasting spirits are also reported by Hallgarten [46]. One participant was, however, virtually perfect at discriminating whisky from cognac (50/51 correct) and virtually perfect at identifying the whisky brand too, while being only just better than chance at discriminating the brandy brands.

More recently, Barry Smith and his colleagues [34] conducted a much more thorough study covering much the same ground. Once again, though, the results revealed how those tested, in this case, people having different levels of familiarity with the blends vs. single malts distinction from the USA, France, and the UK were unable to discriminate reliably, either from nosing or tasting, the blended whiskies from the single malts in a free-sorting task. The participants, both experts and novices (92 in total) were instructed to sort 10 samples, four blended whiskies, four single malts, one repeat, and one single grain whisky. Once again, no information about the drinks that were to be tasted was presented. While the experts were, as one might have hoped, better than the novices when it came to picking out the repeat from amongst all of the samples, none of the participants were able to identify any of the whiskies at a level that was better than chance.

### 2.3. Interim Summary

The blending of wines or whiskies typically has the aim of combining different components in order to deliver a consistent flavour profile year-on-year. This is what one might consider a ‘flavour metamer’ [47] (p. 150). This is certainly the aim when blending non-vintage champagnes. Indeed, the blending of champagnes and ports commonly involves a complex mixture of wines from different vineyards, vintages, and winemaking styles [48].

Here it is perhaps also worth noting that the inability of tasters to segregate figures from the ground in the case of human olfaction has been documented previously in a number of laboratory studies using a wide range of artificial/arbitrary mixtures of odorants and tastants [49,50,51,52]. Indeed, even trained assessors appear unable to pick out more than two or three elements in a mixture of odorants, tastants, or flavours [53]. Intriguingly, though, the situation in humans is very different from what has been documented in mice. The latter are capable of distinguishing one target odorant from a mixture, with the accuracy of their performance declining only marginally (from 94% to 85% correct) as the number of odorants in that mixture increases all the way from 2 to 15 [54,55,56,57].

### 2.4. The ‘Flavour Blending Hypothesis’

One of the explanations that have been put forward to help explain why people might find it so difficult to discern the contributing elements in a mixture of flavours relates to the ‘flavour blending hypothesis’ first articulated by Dubow and Childs [58]. The latter researchers drew attention to the existence of various non-linear mixture perception effects. Indeed, a number of unpredictable mixture suppression and mixture enhancement effects have been reported in the literature over the years under laboratory testing conditions. For example, Stevens and Cain [59] reported that mixtures of tastants may operate in what has been described as an erose and non-monotonic manner. Meanwhile, one everyday example of the sometimes unpredictable consequences of mixing is illustrated by what happens when salt is added to tonic water. First, it becomes sweeter due to the release from masking only to become noticeably saltier as the amount of salt that has been added increases [60]. At this point, it is important to note that when mixed different flavours do sometimes combine to deliver a new emergent flavour experience, one that is not present (or detectable) when the component elements are presented individually [61]. More often than not, though, it would seem that the emergent taste/flavour is simply less desirable, as in the case of the metallic taste that people used to encounter when combining certain red wines with white fish [62,63].

When Dubow and Childs [58] actually tested whether it is possible to move consumers from traditional Coca-Cola Classic through to New Coke in a discrete number of steps, their results supported what they describe as a ‘Gradualist Approach Hypothesis’ instead. That is, no sudden change in perception was documented as the researchers moved from 100% of the old product through to 100% of the new formula in a relatively small number of discrete steps. Such a predictable (i.e., unsurprising) perceptual logic to the combining (or mixing) of two flavourful products is certainly also consistent with Lapid, Harel, and Sobel’s [64] claim that they were able to predict the subjective pleasantness of binary mixtures of olfactory stimuli. That said, it may well be that while certain mixtures/blends operate in such a predictable perceptual manner, others do not. 

Master blenders tend to describe what they do as being as much of an art as a science [36]. As Gogoi [36] (p. 53) says of tea: “Blending is a much highly elevated art form; though not exactly scientific, it’s a highly skilled art practiced by a few who truly know their craft.” Indeed, Laing et al. [65] capture the challenge when, in the preface to their edited volume on the topic of mixture perception, noting that: “Each chapter clearly demonstrates that our knowledge and understanding of mixture perception is in its infancy and that progress will require workers to be aware of and apply the findings of others in different disciplines to their research problems.”

### 2.5. Mixing/Mixed Drinks

Let us return now to the distinction between blending and mixing. To the extent that these terms can be differentiated, mixing would appear to refer to the situation in which quite different products are combined, as in the case of a mixed drink, say. It would seem to imply the components are rather different in kind, whereas blending would seem to imply that the combination of elements are rather more similar to begin with. Mixing often, but perhaps not always, seems to result in the retention of the identifiable contributing elements of the combined stimulus in the mixture. For instance, Kalimotxo, a popular drink in the Basque country, combines cheap red wine with Coke [66]. That said, there have been complaints recently about the incorporation of Sauternes wine into cocktails, the suggestion being that such a ‘perfect’ expression of wine should never be mixed (with what must necessarily be an inferior product) [67]. Meanwhile, over in Hong Kong (and certain other parts of Asia), a mixture of seven parts milky tea to three parts coffee, known as Yuenyeung, is also popular. There are also some rather more unusual mixtures of individually familiar products out there such as, for example, the mixing of whisky and tea, as recommended in an article that appeared in The Wall Street Journal [28]. Tea flavours have also been incorporated into a number of other products, such as, for example, Earl Grey tea-flavoured gin. In the latter case, however, rather than a mixture that has been created, it would appear to be described as a flavouring that is added to the gin [68]. Whether one calls it a mixer, a mixture, or a flavouring would seem, then, to depend, in large part, on the relative amount of the various contributing elements in the mix too.

The interest in mixing distinct elements has recently been extended to the condiments and sauces category too, with the very public launch of ‘Mayochup’ by Heinz providing one such prominent example [69]. Heinz launched an online campaign promising to introduce a new mixture of Mayonnaise and Ketchup (what might be familiar to some as rosemarie sauce, e.g., as once popular for prawn cocktails) should sufficient numbers of the general public vote for the new combination online. Heinz also created something of a stir in the UK recently when they threatened to combine, or mix, baked beans and spaghetti hoops in the same can [70]. 

## 3. Fusion Cuisine

Fusion cuisine is all the rage these days but how, exactly, should it be defined? Stano [26] defines “fusion cuisine” as “a style of cooking combining ingredients and techniques from different foodspheres. Asian fusion restaurants, for instance, offer blends of various cuisines of different Asian countries and the culinary traditions of the places where they have become increasingly popular. Similarly, the Tex-Mex cuisine combines the South-western United States culinary system with the Mexican foodsphere, while the Pacific Rim cuisine is based on the mix of different traditions from the various island nations; and so on and so forth. In all these cases, foods based on one culinary culture are prepared using ingredients, flavours, and techniques inherent to another culture. Consider, for instance, the “Taco Pizza”, made with cheddar and pepper jack cheese, tomato sauce, refried beans and other common taco components.” According to Stano [26], fusion implies “a harmonious combination of different culinary traditions in order to create innovative and seamless dishes”.

At one level, fusion foods can be seen as representing another kind of mixing of distinct elements in food. Indeed, it can be argued that successful examples involve the combination of two or more distinct, yet identifiable, elements/culinary approaches. The last decade or so has seen the much-publicised arrival of a number of very successful fusion foods in the bakery category: First came the ‘cronut’ [9,10], then the ‘mufgel’ [12], and, most recently, the ‘croiffle’ [13]. While some commentators have wanted to compare these new inventions to frankenfoods [71], it is hard to deny their phenomenal appeal to consumers. Though, whether that success owes more to the irresistible taste experience that such fusion foods promise or to the trend-setters wanting to stay ahead of the curve in terms of spotting the next trend and, more importantly, Instagramming about is, I would argue, rather harder to say [14,15].

It is all too easy to think of the interest in new fusion foods as an exclusively contemporary phenomenon, given fusion cuisine’s current popularity in the media. However, it should be remembered that fusion cuisine has long been popularized by the likes of chef/restaurateur Wolfgang Puck [72]. At the same time, however, many of the everyday foods that we are all familiar with technically count as fusion foods, at least if one goes back far enough in the historical record [73]. Take a suitably long-term view, and it soon becomes clear that most of the foods that we eat today actually represent a fusion of ingredients, flavours, components, recipes, styles, and/or food philosophies (see [2] for a number of examples).

### Confusion Cuisine

Stano [26,27] has written a number of insightful papers around the theme of ‘con-fusion’ foods. Her suggestion is that novel food combinations do not always give rise to a successful new fusion food/product but may instead result in con-fusion. As Stano [26] notes: “…fusion cuisines run the risk to degenerate into “con-fusion cuisines”, causing inevitable clashes between incompatible flavours and textures, and fomenting a chaotic overlapping between different foodspheres and “food identities”.” Such ‘con-fusion’ foods are likely to result when the consuming public cannot read the signs correctly.

There is undoubtedly a widespread contemporary interest in fusion foods, especially amongst food marketers and the consuming public at large. The latter’s ‘hunger’ for the many putatively new fusion foods introduced into the food marketplace in recent years can perhaps be framed within a broader interest in all things novel, and experimental, in the world of cuisine (see the earlier discussion on this point). That said, it is important to remember that coming up with a genuinely new fusion food, food product, or dish, is not as easy as it might at first seem. New fusion cuisine can, then, all too easily end-up leading to ‘con-fusion’, if the consumer doesn’t know how to ‘read’ the new culinary creation. This is why getting the name right (i.e., flagging the discrete elements that have been fused) can help the consumer to decide where exactly the innovation lies in a putatively new product.

## 4. Flavour Pairing

There has been an explosion of interest in the topic of flavour pairing in recent years [31]. It is interesting to note here how what was traditionally restricted to something that one might come across with a wine pairing at a fancy restaurant has now been extended to a wide range of pairings [29,30], including everything from beer and food [74,75], tea/coffee and cheese/chocolate [76] all the way through to even more esoteric pairings. Although falling beyond the scope of the present review, it is worth noting that Spence [31] has recently argued that the majority of flavour pairing principles that have been articulated by those working in the field can, in fact, be reduced to a number of perceptual pairing principles (see Table 1).

One finds researchers suggesting that choosing effective food-beverage pairing (just like the art of blending mentioned earlier) currently remains as much an art as a science [77,78]. Indeed, according to Maresca [79] (p. 7): “Success in wine and food matching depends on nothing more abstruse than finding out why certain foods and wines affect each other for good or for ill and learning how to generalize from that simple information to predict the way other wines and food will interact”. This despite the attempts by those who have wanted to promote the flavour pairing hypothesis (FPH) [80,81,82]. Indeed, the emerging field of computational gastronomy builds on the FPH: “If two ingredients share important flavour compounds, then they will go well together.” [80]. Or take de Klepper’s [83] (p. 55) slightly more nuanced definition: “The more aromatic compounds two foods have in common, the better they taste together. This effect is particularly strong when two foods share aromas that make up their characteristic flavour.” 

World-famous chef Heston Blumenthal was initially a vocal proponent of molecular flavour pairing. As Ahnert et al. [81] (p. 2) notes: “The chef Heston Blumenthal, together with flavour scientists has suggested that two foods that share chemical flavour compounds are more likely to taste good in combination.” Unfortunately, however, although popular, the FPH simply does not work in terms of predicting those combinations of flavours or ingredients that will necessarily pair well together [83,84]. For, contrary to the claim made by Jain, Rakhi, and Bagler [85] (p. 3) that: “Molecular composition of food dictates the sensation of flavour”, it would appear that matters are, in fact, much more complex than a simple chemical analysis would predict. As top chef, Heston Blumenthal [86] (pp. 171–172), once put it: “I soon realised that the molecular profile of a single ingredient is so complex that even if it has several compounds in common with another, there are still as many reasons why they won’t work together as reasons why they will… Molecular profiling is a great tool for creativity, but it supports intuition, imagination and emotion rather than replacing them.”

Chef Blumenthal subsequently went further, noting that coming up with effective flavour pairing is actually much more challenging than might be suggested by the food-pairing hypothesis: “Looking back at my younger self I’m almost embarrassed at my bumptious enthusiasm, not least because I now know that a molecule database is neither a shortcut to successful flavor combining nor a failsafe way of doing it. Any foodstuff is made up of thousands of different molecules, that two ingredients have a compound in common is a slender justification for compatibility. If I’d known then what I know now, I would probably never have tried this method of flavor pairing: there are simply too many reasons for it not to work. As it was, in my naivety I just got stuck in.” [87] (p. 45).

Flavour-pairing therefore currently remains as much an art as a science. That being said, not everyone is convinced that all the excitement around flavour pairing is necessarily warranted. For instance, just take the following quote from Mike Steinberger [88], writing in the online magazine Slate: “If one were to compile a list of the least-significant issues confronting mankind in the first decade of the 21st century, the question of wine’s compatibility with cheese would surely rank high.” A similar sentiment was expressed by Ferran Centelles [89], former head sommelier at the El Bulli restaurant, writing on Jancis Robinson’s website that: “I keep recalling the realistic statement Jancis made during the International Culinary Forum in Barcelona in September 2012: ‘we would be sending out a very negative message if we gave people the impression that finding the perfect pairing is terribly important and that something would go wrong if you just drink what you wanted and ate what you wanted’. I could not agree more with that. I strongly believe that matching is overvalued on many occasions and sometimes it receives exaggerated attention from consumers and professionals.”

At the same time, however, there can be little doubting just how much the idea of flavour pairing has caught the public’s imagination in recent years [31,90]. As we will see in a moment, the notion of pairing elements in a tasting experience has now gone beyond the pairing of flavourful stimuli to the crossmodal pairing of flavour stimuli with sound, as well as with other sensory elements, such as colours, shapes, and textures.

## 5. Interim Summary

On the basis of the literature review that has been outlined thus far, it would appear that a wide variety of different perceptual outcomes are possible when different ingredients, flavours, and/or culinary techniques are combined in a dish, drink, food product, or multi-element tasting experience. While there continues to be interest in the next new food experience, after the most obvious global cuisines have been sampled, the next thing that creative chefs/flavourists are increasingly trying to do is to combine elements in a product, dish, or drink in a novel manner, in the hope that they will end-up capturing the public’s imagination. While the aim is sometimes fusion (i.e., to create perceptual metamers from mixing components whose individual identity are hard to discern [47]), the growing commercial and academic interest around fusion cuisine/food [2] and flavour pairing [31,63]; though see [88,89], for a couple of dissenting voices) can also be seen as providing another means of combining flavour sensations in an innovative manner. 

However, while many examples of fusion foods have proved very popular with the public at large [2], there is always a danger of ending up with confusion instead [26,27]. As Stano stresses, one can avoid the confusion in fusion foods by making sure that the individual elements are recognizable once combined. What is more, it is also worth bearing in mind here just how many of the foods we like nowadays turn out, on closer inspection, to be fusion dishes [72]. When considering the blending, mixing, fusion, and pairing of ingredients, flavours, and/or culinary techniques, one should also be sensitive to the danger of presenting something that is too complex. Though, that said, it is perhaps surprising how rarely in the world of food and drink one comes across flavour experiences that are described as being ‘overly complex’ [24,91].

Having reviewed the literature on combining various elements from within the chemical senses (resulting in blending, mixing, fusion, confusion, and flavour pairing), we are now in a position to address the question of pairing sensations once again, though this time round, we will do it from a more crossmodal perspective. In particular, in the sections that follow, I want to review the emerging literature on the pairing of flavourful stimuli with sound. 

## 6. Pairing Flavours with Music and Soundscapes

Having studied the pairing of flavourful elements, be they ingredients, techniques, or culinary styles, the next question that I wish to address, in the second part of this review, is the pairing of flavourful elements with stimuli presented in a different sensory modality. Initially popularized by the Italian Futurists in the 1930s [92,93], the last couple of decades has seen a veritable explosion of interest in the pairing of tastes, aromas, oral-somatosensory food attributes (such as creaminess [94]), trigeminal stimuli (such as spiciness [95]), and flavours with a range of seemingly-unrelated stimuli presented in other sensory modalities [96,97]. While often talked of as a kind of synaesthesia [38,98,99], these crossmodal correspondences have now been documented between components of flavourful stimuli on the one hand, and colours [100,101], shapes [102,103,104,105], textures [106,107,108,109], and sound stimuli [110,111,112], on the other. Note here that crossmodal correspondences are defined as the often surprising associations that exist between different stimuli, attributes, or dimensions of experience in one sensory modality and those in another sensory modality, no matter whether physically present, or else merely imagined [96,113]. In contrast to synaesthetic associations, which are also surprising, crossmodal correspondences tend to be shared by the majority of people [114]. Synaesthesia, by contrast, is defined by idiosyncratic mappings between the inducer and the concurrent stimulus [115].

In the remainder of this paper, I wish to focus specifically on the pairing of flavourful stimuli with music or soundscapes. In part, this is simply because this particular pairing of sensory inputs has seemingly attracted far more research interest than any of the other modality pairings that were just mentioned [116]. Perhaps more importantly, though, the temporally-evolving nature of both musical compositions and carefully crafted complex flavour experiences (such as when tasting coffee, wine, cognac, whisky, or chocolate [117,118,119,120]), means that there may also simply be more scope for perceptual interactions (and crossmodal matching) than is the case for time-invariant, colour, shape, or texture stimuli. In general, this new interest in pairing apparently-unrelated sensations (specifically, sound and flavour stimuli) goes by the name of ‘sonic seasoning’ [121].

### 6.1. Semantic Pairing of Music and Food/Beverage Stimuli

There is a long history of semantic priming research showing, for example, that playing French music biases people toward choosing French (over German) wine in the supermarket, the latter chosen more often when people heard German music instead [122,123]. Meanwhile, in another study, playing Flamenco-like Spanish music was found to bias people toward choosing paella rather than Italian food in a North American canteen [124]. By now, there is extensive evidence that across a range of venues, the music that happens to be playing in the background will likely bias our choices, no matter whether we realize it or not, and mostly the evidence suggests that we do not [93,116]. There is also some limited evidence for the semantic attributes of music, or the emotional/conceptual associations, to carry over and so bias what people say about the tasting experience and how much they enjoy it ([125,126]; see also [127]). There is, for example, evidence to suggest that playing classical music may also prime notions of quality/class. Indeed, it has often been noted that people spend tend to spend more on food and drink when classical music happens to be playing in the background [128,129]. Note that this kind of approach to pairing would seem most similar to the conventional approach listed under the Cognitive/Intellectual stream of the main flavour pairing principles listed in Table 1.

Crossmodal (priming) influences of pleasant music on the tasting experience have also been demonstrated [130]. Put simply, the more you like the music that happens to be playing, the more you will report that you like the tasting experience that happens to have been paired with it. However, specifically in terms of matching, or pairing, one might think of the emotional mediation that has been shown to explain a good part of the literature on crossmodal correspondences. In particular, when it comes to the crossmodal mapping, or pairing, of flavourful stimuli, numerous studies have documented a robust mediating role for emotion [131,132]. It is important to note that while emotion cannot explain all the variance, it often comes up as a major explanatory variable in terms of these and other crossmodal correspondences.

### 6.2. Oenesthesia

This is the name given by Jo Burzynska, a New Zealand wine judge and sound artist, to those examples of sonic seasoning that focus on the crossmodal mappings that involve wine [38]. Indeed, when thinking about music’s influence on the wine-tasting experience, it can be helpful to discriminate between four different kinds of judgments, or impressions that we may ascribe to a wine [112]: Hedonic—how much do we like the wine? Sensory—our assessment of the physical properties of the wine (such as its sweetness, acidity, alcohol) and their impact on the drinker (astringency, length, etc.); Analytic—concerning such attributes as age, complexity, balance, quality, and price assessment; and Descriptive—would one describe the wine as heavy or light, zingy or lush, masculine or feminine? Music can potentially be paired with, and hence influence all four kinds of judgments. Indeed, by now, all four have been shown to be susceptible to the influence of musical interventions. That said, the question of whether they are all equally susceptible to such crossmodal influence is still an open one, and anyway hard to address unequivocally. More conceptual associations/priming related to the harmony and the art of blending have also been used. Here, one might think only of the sensory app and orchestral events put on by the Krug champagne brand [133].

There are, then, a numerous conceptual, as well as more perceptual, reasons for wanting to pair music with flavourful stimuli crossmodally. It is at this point that it may be worth revisiting the food and wine pairing quote by Maresca [79] mentioned earlier since it can be easily rephrased to explain what may be going on in the case of wine-music matching: “Success in wine and music matching depends on nothing more abstruse than finding out why certain pieces of music/musical parameters and wines affect each other for good or for ill and learning how to generalize from that simple information to predict the way other wines and music will interact”. Next, I would like to focus specifically on crossmodal perceptual pairing.

### 6.3. Pairing of Music and Food/Beverage Stimuli Based on Perceived Similarity

At this point, it is important to stress the fact that a number of so-called crossmodal sound-flavour correspondences may not necessarily be based on the perceived similarity between the auditory and flavourful stimuli. Rather, on occasion, pairs of stimuli that are presented in different sensory modalities are associated because one of the stimuli predicts the other, much in the way that the sound of the bell led Pavlov’s dogs to expect their food, say [134]. Indeed, one might consider the crossmodal associations between colour and basic taste, or flavours, in much the same light [100,135]. That is, people may well associate a pinkish-red colour with sweetness not because they perceive the component stimuli to be similar, but rather because, in the absence of any other information, they expect pinkish-red foods to taste sweet, as a result of a learned association [101,135,136,137]. It is an open question as to whether such crossmodal predictive coding [138,139] should necessarily be labelled as a kind of crossmodal correspondence or not. Those who wish to emphasize the perceptual similarity that many historically have stressed as being core to the correspondences [140,141,142] would likely disagree.

By contrast, those who take correspondences to be surprising connections between the senses that may influence behaviour, and are based on the pick-up of amodal or object-specific sensory pairings (as when we match the bark with the image of a dog) would presumably be keen to include them. Indeed, some have argued against the very idea of crossmodal similarity. For instance, at the end of the 19th century, Helmholtz, the eminent early psychophysicist, had the following to say: “The distinctions among sensations which belong to different modalities, such as the differences among blue, warm, sweet, and high-pitched, are so fundamental as to exclude any possible transition from one modality to another and any relationship of greater or less similarity. For example, one cannot ask whether sweet is more like red or more like blue. Comparisons are possible only within each modality; we can cross over from blue through violet and carmine to scarlet, for example, and we can say that yellow is more like orange than like blue!” [143] (p. 77).

At present, it is certainly hard to come up with any account for the crossmodal correspondences between flavourful stimuli and the perceptual attributes of musical stimuli that are based on predictive coding or learned associations (though see [97] for one postulated source for the pitch-basic taste correspondence). Rather, the majority of the mappings that have been reported to date would appear to be based firmly on fundamental perceptual similarities instead. Indeed, it is noticeable how many of the same terms are used to describe both olfactory and musical stimuli—think only of ‘low notes’ and ‘high notes’, ‘chords’, and ‘harmonies’, all terms, note, that can equally well be used to describe auditory and olfactory stimuli [144,145]. According to some, it is the shared linguistic terminology that may underpin the correspondence, and hence congruent pairing [146]. However, it is also important to stress here how the shared language may itself be picking-up on a more fundamental perceptual similarity [147].

### 6.4. Pairing of Music and Food/Beverage Stimuli: A Practical Perspective

The notion that music or soundscapes might be deliberately paired with a tasting experience is one that has undoubtedly grown in popularity in recent years. Several chefs/restaurants have already started to incorporate atmospheric environmental soundscapes in order to complement the food that is served. One of the first examples here was the “Sound of the sea” dish served at the three Michelin-starred The Fat Duck restaurant in Bray, UK [86,148]. For one of the courses on the tasting menu, diners are encouraged to insert a pair of earbuds prior to starting the dish, whereupon they will hear a carefully crafted soundscape incorporated the sound of seagulls and the waves crashing gently onto the beach. The dish has been the signature dish on the restaurant menu for more than a decade, now. It emerged out of research conducted together with the chef and his team showing that pairing oysters with a seaside soundscape led people to rate the oyster as tasting significantly better, but no more salty, than when the oyster was sampled while listening to a different soundscape instead [149]. Elsewhere, chef Jozef Youssef of Kitchen Theory pairs several of the dishes on his tasting menu with a variety of matching soundscapes and sound effects that incorporate matches based on everything from a semantic match through to crossmodal correspondences [150,151,152]. While it will clearly not be appropriate/easy to offer customers such sound–flavour pairing experiences in a regular (e.g., casual) dining context, it is interesting to see how the approach has now started to spread more widely.

**Figure 1:**
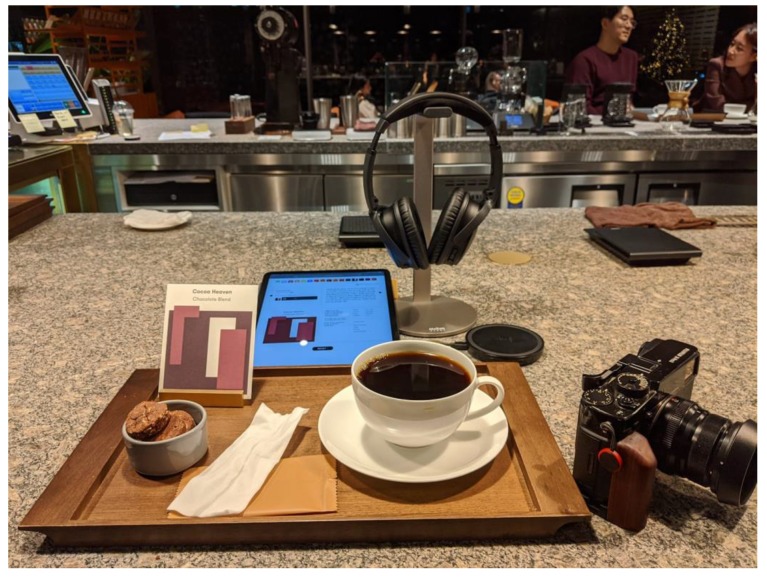
Korean coffee-shop where music is paired with the choice of coffee designed to match the customer’s taste preferences.

**Figure 2:**
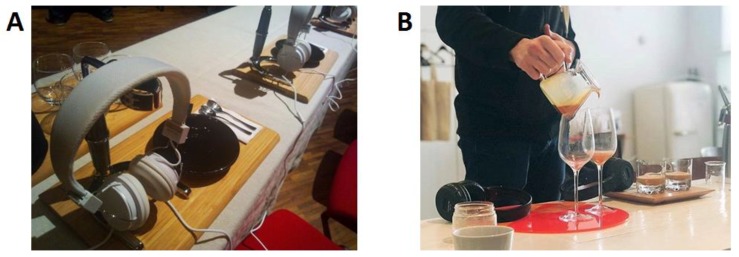
Multisensory experience designs during barista championships. (**A**) Rasmus Helgebostad’s sonically-enhanced coffee drink served as part of his entry in the 2011 Norwegian barista championships; (**B**) Matt Winton’s multisensory experience of the same signature coffee drink being served in two different setups (including distinct soundtracks) in the 2018 World Barista Championship [155].

For instance, one Korean coffee shop has recently started inviting its customers to “fill in a form” (on an iPad) regarding their taste preferences [153]. The app then selects the best-matching coffee blend and musical selection (see Figure 1). Finally, you are served a coffee with a pair of wireless headphones, in order to deliver a genuinely multisensory tasting experience [154]. Meanwhile, at the high-end of multisensory coffee experience design, top baristas such as Rasmus Helgebostad and Matt Winton made a sonically enhanced coffee drink a few years ago as part of their barista championships routines (see Figure 2).

Elsewhere, in more of a marketing-led intervention the Xin café in Beijing used augmented glassware to play sweet music and so reduce the sugar content in the drinks they served ([156] see also [157]. However, the much more widespread exposure to flavour–music pairing has emerged through branded experiential events and sensory apps [158,159,160,161,162]. So, for example, champagne house Krug, launched its Music Pairing series a few years ago [163,164]. With this app, the consumer can scan their wine label, or else type the individual number appearing on the back of their bottle, in order to access a selection of music that has been chosen to match the specific wine. “Krug’s “Music Pairing” has recording artists select their picks for tracks to accompany six particular varieties of Champagne, much like a chef would note what food pairs best with a particular wine” [164]. At present, the musical matches that are offered are idiosyncratic, as highlighted by the following quote from Argentine pianist, Marcela Roggeri: “There are certain champagnes that I associate only with classical music like Krug Clos du Mesnil or Krug Clos d’Ambonnay. However I could associate other Krug champagnes with jazz or Brazilian music, such as Krug Rosé.” [163].

Meanwhile, Stella Artois teamed up with the pop group The Roots, experience designers, Bompas and Parr, and myself to create a special music video in 2016 as part of Le Savoir, a multisensory entertainment platform [165]. In this case, the idea was that people sitting at home might enjoy a drink of beer with the paired music video. Simply moving the cursor while watching the specially composed track and associated music video (called “Sweet to the Bitter End”) allowed the consumer to bring out a sweeter (fruitier) or a more bitter version of the instrumentation/video backdrop. The suggestion that this personalized version of sonic seasoning could then be used to adjust the drink to taste—a very literal form of digital seasoning. The online activation was also associated with a series of experiential dinners. Todd Allen, VP of Global Marketing at Stella Artois reported that: “It’s bringing millennials’ passion points of food, music and art together under one platform to deliver an immersive dining experience, all perfectly paired with Stella Artois…We’re very excited to bring it to the market.” [165]. Ultimately, however, it may well be generic sensory apps that may offer this service to us, as already occurs with one app that promises to provide a matching wine if you scan the label of the wine bottle [166].

### 6.5. Why Bother Pairing Music/Soundscapes with Food/Beverage Stimuli?

Finally here, it is interesting to note that there is growing interest not just in modifying taster’s ratings of food and beverage, such as, for example, fruitiness, acidity, or sweetness, but in actually delivering extraordinary tasting experiences that are somehow more (or greater) than the sum of their parts [167,168]. There have, for instance, been occasional reports of people being brought to tears by the combination of wine and purposely-composed matching music [169]. Elsewhere, one finds descriptions such as the following from James John, Director of the Bath Wine School, talking about tasting Chardonnay while listening to Mozart’s Laudate Dominum: “[…] Just as the sonant complexity is doubled, the gustatory effects of ripe fruit on toasted vanilla explode on the palate and the appreciation of both is taken to an entirely new level” [170]. The possibility of delivering such extraordinary multisensory tasting experiences by carefully combining (or pairing) music and tasting providing one answer to the refrain that is sometimes heard (especially, it would seem, from Masters of Wine) concerning why one should bother with changing the taste of, for example, wine via musical accompaniment when one could, for instance, just pick a different wine in the first place [112]. In a similar vein, we saw earlier, there were some commentators who did not think that flavour-pairing was all that important as far as the chemical senses are concerned, or to be more precise, at least not that important in the case of food and wine matching [88,89].

## 7. Conclusions

In conclusion, in the first part of this review, we reviewed the various ways in which the elements in a tasting experience can be combined. When considering the combination of two or more distinct flavourful stimuli, the desired results may be in terms of blending, mixing, or fusion (confusion in the less successful cases). However, there has also been a growing interest in flavour pairing. It has been argued that all examples of successful flavour pairing are based either on a cognitive/intellectual matching, or else can be reduced to one variant of a perceptual match. Various perceptual outcomes are possible when flavours are paired, including, Similarity; Contrast; Harmony; Emergence; or Modulation (either Suppression or Enhancement). In the second part of the review, the focus was on crossmodal pairing involving flavour as one of the component stimuli. The focus here has been specifically on two closely related senses, namely pairing flavours with music and soundscapes, based on the logic of crossmodal correspondences—the surprising similarities that we experience between seemingly-unrelated sensations in different sensory modalities.

There is undoubtedly growing commercial and academic interest in this kind of crossmodal pairing, colloquially known as ‘sonic seasoning’. It is an intriguing question for future research to determine whether all of the perceptual pairing principles identified in the case of flavour–flavour pairing can be extended to the crossmodal pairing of flavours with sounds. As we have seen there is already good evidence for a number of such crossmodal associations, and, beyond that, building on these associations to pair music/soundscapes with flavourful stimuli in order to enhance the multisensory tasting experience [171,172,173,174,175,176]. By so doing, it will hopefully be possible to continue to feed the continuing craving for novelty. Particularly exciting recently has been the emergence of work where the pairing of matching of music to flavour is matched over the temporally-evolving flavour experience [177]. It is further intriguing to see how a number of food artists and designers have also been exploring the multisensory interface between audition and flavour experience [178,179,180,181,182].

## Figures and Tables

**Table 1:** Approaches to flavour pairing according to Spence [31]. This table highlights the suggested division of food-beverage pairing approaches into two main categories, with examples of each, and relevant comments, where appropriate.

Paring Approach	Specific Approach	Notes
Cognitive/intellectual		
	conventional	By far the most comments approach to pairing
	complexity	Plached as cognitive/intellectual Category on the assumption that complexity cannot be directly perceived
	quality	Placed as cognitive/intellectual category on the assumption that quality cannot be directly perceived
	process	E.G., pairing wine and cheese because both reply on fermentation
	Shared molecules	While the FPH put forward as a means of predicting perceptual similarity, its failure means that FPH can only meaningfully exist as a cognitive/intellectual reason to pair elements
Perceptual		
	similarty	This approach to pairing is addressed by the FPH
	Contrast	
	Harmony	
	Emergence	
	Modulation-suppression	Typically this approache to pairing involves the suppression of an undesirable elements in the tasting experience
	Modulation-enhancement	

FHP: Flavour pairing hypothesis.
